# Chromosome Duplication (14q) and The Genotype Phenotype Correlation

**Published:** 2014-03-09

**Authors:** Ariane Sadr-Nabavi, Morteza Saeidi

**Affiliations:** 1 Department of Medical Genetic, Faculty of Medicine, Mashhad University of Medical Sciences, Mashhad, Iran; 2 Iranian Academic Center for Education, Culture and Research (ACECR), Mashhad, Iran; 3 Medical Genetic Research Center (MGRC), School of Medicine, Mashhad University of Medical Sciences, Mashhad, Iran; 4 Department of Neurology, Mashhad University of Medical Sciences, Ghaem hospital, Mashhad, Iran

**Keywords:** Chromosome Duplication, Mental Retardation, Chromosome 14

## Abstract

The rearrangement of chromosome 14 is a rare cytogenetic finding. Changes in the number or structure of chromosome 14 can have a variety of effects, such as delayed growth and development, and distinctive facial features. The human chromosome 14 plays an important role in imprinting events importunes of a structural rearrangement is specifically when a phenotype is caused by imprinting, whereby the interpretation of genotype-phenotype correlation becomes extremely difficult. In this study, we examined a 3 year-old mentally impaired girl with unusual facial features. G-banding showed terminal duplication of chromosome 14 in the karyotype of the patient. In this particular case, we explained a phenotype genotype correlation in a patient with a dup (14) rearrangement, thus emphasizing the importance of prenatal diagnosis for pregnancies with an abnormal nuchal translucency.

## Introduction

A chromosome anomaly can be (a) Numerical: there is one (or more) chromosome(s) in excess (trisomy) or missing (monosomy) resulting in the karyotype being always unbalanced and (b) Structural: the change is balanced, if there is no loss or gain of genetic material but unbalanced, if there is deletion and/or duplication of chromosome segment(s). In an unbalanced chromosome rearrangement, the chromosomal complement contains an incorrect amount of chromosome material and the clinical effects are usually serious. Duplication is one of the structural changes that results in an unbalanced rearrangement. There are two types of duplication: 1. Direct: segment of chromosome is repeated, once or several times, the duplicated segment keeping the same orientation with respect to the centromere ("tandem duplication") and 2. Inverted: the duplicated segment takes the opposite orientation (1, 2). 

Chromosomes in the human genome is the chromosome 14, the short arm of this chromosome is characterized by heterochromatin which contain ribosomal RNA genes. The long arm of this chromosome is euchromatin that most of the geneslocatedon , is the protein-codinggenes (3) . About 106 million base pairs (bp) DNA building blocks spans the chromosome 14. This is approximately 3.5 percent of the total DNA in the cells and contain between 800 and 1,300 genes (4). 

The chromosome 14 is one of the human chromosomes known to have an imprinting affect. In the human chromosome, 14-uniparental disomy (UPD) describes the inheritance of both the homologs of a pair of chromosomes from an individual parent. Though genomic imprinting is correct in UPD, problems arise due to the normal chromosome segregation, therefore causing false imprinting. The phenotypes have been described for maternal UPD 14 respectively (5-10).

Paternal UPD 14 is associated with developmental delay. Both maternal UPD 14 and paternal UPD 14 rarely appear in individuals . Here we describe the clinical features associated with a dup (14) observed in a 3 year-old girl. G- banding was used for detailed evaluation of the chromosomal gains and losses.

## Case report

 In a cousin marriage between a 29 year old female and a 35 year old male, following a 15 hours prolonged labor, a child was born by caesarean section. The infant’s weight, height and head circumference at birth were 2200 g, 46 cm and 31cm respectively. The result in biochemistry was creatine 0.4 mg/l, in hematology HCT 25%, platelet 530000, PT 16.5 sec. PTT 36 sec and INR 1.5. Overtime the mother noticed delay in the growth and development of the child and opted for a different workup. In the new laboratory assessments , the following findings were observed : Zinc: 59 mcg/dl (N: 63.8-110), Sweat test: (weight 529 mg, Cl- 20, Na+ 20), ABG: pH: 7.38, PO2 : 33.5 mmHg, HCO3 -: 19.9 mmol/l, PCO2 : 33.2 mmHg and O_2_ Sat: 63.1%.

**Fig 1:**
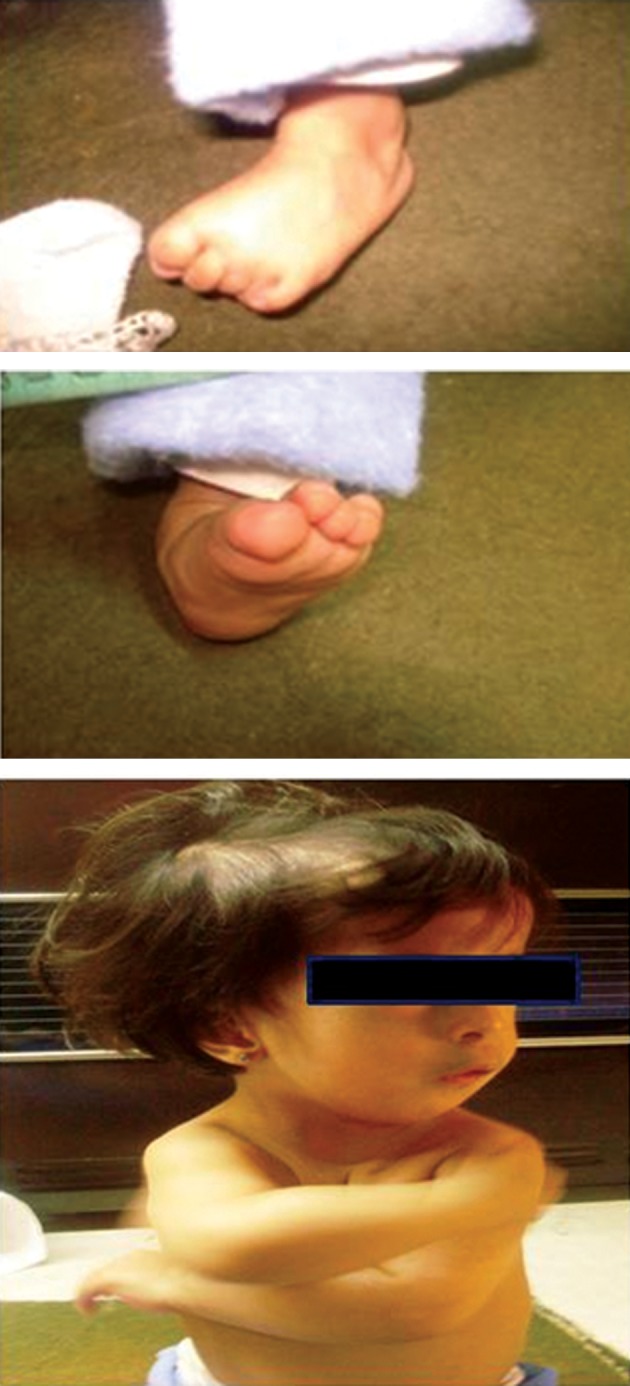
Examination revealed evidence of generalized weakness with reduced muscle tone and diminishment. Patient was unable to walk unassisted and also had difficulty sitting.

Following abnormal ABG and mild cyanosis in the patient, echocardiography was carried out and the results were PDA, ASD20size 7-8mm, L->R shunt, good ejection fraction and no pulmonary hypertension. The patient underwent surgery via Video-Assissted Thoracoscopy (VATS) and Ductus which was closed using double liga clips. Since then her weight loss has continued. She received hormone therapy which was rendered ineffective and finally a genetic consultation was carried out. The patient was admitted to the Imam Khomeini Hospital in Tehran on 03.09.2010 at the age of 34 months. At present, the girl’s weight and height are at 7100 g and 54 cm respectively. She has gained the ability to sit and as of lately can manage a few one-syllable words. She suffers from skin allergy, insomnia and recurrent gastroenteritis and is undergoing physiotherapy and occupational therapy. The patient was diagnosed to be mentally impaired with an unusual facial feature, including a high forehead, epicanthic folds, large and low set ears, a small jaw and chin and also a large tongue. In her lower extremities, overlapping toes was noted. A neurological examination revealed evidence of generalized weakness accompanied with reduced muscle tone, diminished deep tendon reflexes and some diatonic features in the distal parts of the extremities. Due to muscle weakness in the lower limbs, the patient was unable to walk unassisted and also had difficulty in sitting (Fig 1). 

**Fig 2:**
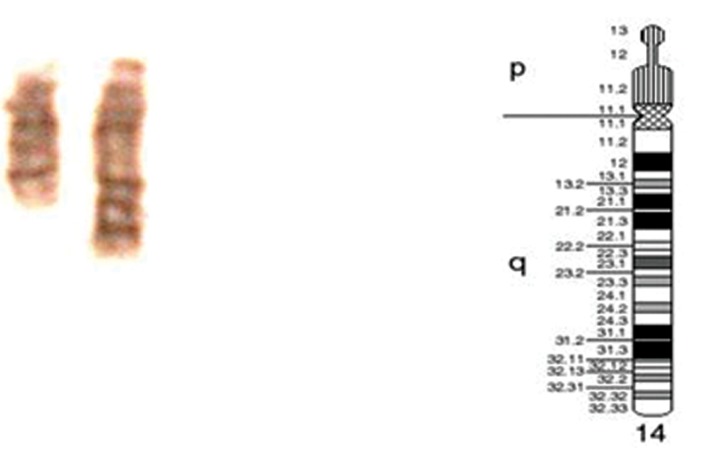
Banding of chromosome 14 [dup (14)].

Conventional cytogenetic analysis of cultured lymphocytes was performed. Blood samples were obtained through venipuncture and collected into heparinized syringes. For each 2 subject, three lymphocyte cultures were usually set up according to conventional techniques. Cultures were made in Ham’s F10 (Biochrom) medium supplemented with LymphoGrow (100ml; Complete medium 12% newborn calf serum, 7.8 μg/ml Phytohaemagglutinin (PHA, CytoGen, Germany), LymphoGrow II (100ml; Complete medium 14% newborn calf serum, 8.8 μg/ml phytohemagglutinin) (CytoGen, Germany). The cells were grown at 37˚C for 48 to 72 hours. Cultures were treated with colchicine (10 μg/ml) (Life Technologies/Invitrogen) during the last 3hrs of incubation. Cultures were harvested using protocol, including hypotonic treatment of 0.56% KCl (0,065 M) (Merck/ VWR) for 20 minutes at 37˚C and three periods of fixation in methanol: glacial acetic acid (3:1). Flame-dried slides were prepared and stained by Giemsa technique. Cytogenetic analysis revealed a 46, XX, dup (14) karyotype (Fig 2). The rearrangement was present in all the 25 analyzed cells. Although the breakpoint was difficult to assign, the G-banding displayed duplication of chromosome 14 at region 2 and band 4. The resolution of the banding was approximately 350 bands. Karyotype analysis of the parents signifies that a de novo rearrangement has occured in this particular patient.

## Discussion

It seems that the chromosomal aberration provides a good explanation for the clinical features of our patient. Cytogenetic analysis revealed a 46, XX, dup (14) karyotype (Fig 2). Transmitted duplications are often slightly less reported than deletions. Glass et al. (11) list parent-to-child transmission of duplications of chromosome 7p, 8p, 9p, 14q and 15q that have been listed in the literature. Since the breakpoint of this duplication was not clear, the exact duplication region of chromosome 14q was not specified. With high resolution G-banding. The resolution of the banding was approximately 350 bands. The general phenotype of the patient was growth and mental retardation, dystrophic features and muscle weakness. This phenotype has been also seen in a patient with chromosome 15 anomalies (12). The human chromosome 15 is known to have an imprinting effect. The phenotypic consequence of genomic imprinting may result from one of the two mechanisms: overexpression of a parent-specific transcript or absence of a parent- specific transcript. Examples of an overexpression of a parent-specific transcript include the imprinted genes involved in Beckwith- Wiedemann syndrome (13) and Russell-Silver syndrome (14).

Examples of absent parent-specific gene expression include brain specific expression of UBE3A in Angelman syndrome (15) and most likely the gene(s) involved in Prader-Willi syndrome (16). As for chromosome 14 and UPD, either mechanism is possible. However, in our opinion based on the comparison between the phenotypes of UPD 14 cases and chromosome 14 duplication cases, the sense of a parentspecific transcript (functional trisomy) results in the phenotypes being associated with maternal and paternal UPD 14. Furthermore, the genes mapped to chromosome 14q are among similar genes encoding placental growth factor (14q24.3), neuroglobin (14q24.3), creatine kinase (14q32) and ataxin3 (14q21) which play major roles in carnal and mental evolutions. Therefore, the duplication of this region could result in the associated phenotypes (17-20).

The present study reports a chromosomal duplication syndrome. Our results suggest that genetic counselling and a follow up karyotyping should be performed when an increased nuchal translucency is observed.
